# Radiological Features for Frailty Assessment in Patients Requiring Emergency Laparotomy

**DOI:** 10.3390/jcm11185365

**Published:** 2022-09-13

**Authors:** Katarzyna Kołodziejska, Jan Witowski, Piotr Tylec, Anna Grochowska, Natalia Przytuła, Maciej Lis, Michał Pędziwiatr, Mateusz Rubinkiewicz

**Affiliations:** 1 Department of Medical Education, Faculty of Medicine, Jagiellonian University Medical College, 30-688 Kraków, Poland; 2 Department of Radiology, New York University Grossman School of Medicine, New York, NY 10016, USA; 3 Faculty of Medicine, Jagiellonian University Medical College, 31-008 Kraków, Poland; 4 Department of Radiology, Jagiellonian University Medical College, 31-008 Krakow, Poland; 5 2nd Department of General Surgery, Jagiellonian University Medical College, 31-008 Kraków, Poland

**Keywords:** frailty, emergency surgery, emergency laparotomy, elderly, osteopenia, sarcopenia, sarcopenic obesity, abdominal aorta calcification rate, renal volume, BGA score, mFI, modified frailty index, brief geriatric assessment

## Abstract

Introduction: As the number of elderly patients requiring surgical intervention rises, it is believed that frailty syndrome has a greater impact on perioperative course than on chronological age. The aim of this study was to evaluate the efficacy of various imaging features for frailty assessment in patients undergoing emergency laparotomy. Methods: The study included all patients that qualified for emergency surgery with preoperative CT scans between 2016 and 2020 in the Second Department of General Surgery. Multiple trauma patients were excluded from the analysis. The modified frailty index and brief geriatric assessment were used in the analysis. CT images were reviewed for the assessment of osteopenia, sarcopenia, sarcopenic obesity, renal volume and abdominal aorta calcification rate. Results: A total of 261 patients were included in the analysis. Multivariate logistic regression identified every next ASA class (OR: 4.161, 95%CI: 1.672–10.355, *p* = 0.002), intraoperative adverse events (OR: 12.397, 95%CI: 2.166–70.969, *p* = 0.005) and osteopenia (OR: 4.213, 95%CI: 1.235–14.367, *p* = 0.022) as a risk factor for 30-day mortality. Our study showed that every next ASA class (OR: 1.952, 95%Cl: 1.171–3.256, *p* = 0.010) and every point of the BGA score (OR: 1.496, 95%Cl: 1.110–2.016, *p* = 0.008) are risk factors for major complications. Conclusions: Osteopenia was the best parameter for perioperative mortality risk stratification in patients undergoing emergency surgical intervention. Sarcopenia (measured as psoas muscle area), sarcopenic obesity, aortic calcifications and mean kidney volume do not predict poor outcomes in those patients. None of the radiological markers appeared to be useful for the prediction of perioperative morbidity.

## 1. Introduction

As life expectancy around the world is increasing, the number of elderly patients requiring surgical intervention rises. Geriatric patients usually have more comorbidities, which increases the risk of perioperative complications [1,2]. Having said that, metrical age itself does not seem to be an independent risk factor for morbidity [3]. Thus, research on risk assessment is shifting its focus to patient frailty evaluation [4]. There are several methods to estimate frailty, including scales that score patients based on the occurrence of different concomitant diseases [5,6]. This approach assumes full access to a patient’s medical records, which creates a serious limitation in emergency surgery situations [7]. Inability to properly communicate with the patient and missing information about their coexisting conditions makes comorbidity scoring ineligible. This is the reason why alternative methods of frailty assessment might be useful in such cases [8].

Several new approaches for frailty assessment using computed tomography (CT) measurements were proposed. For instance, assessment of sarcopenia by measuring psoas muscle area has been proven to be efficient [9,10,11,12,13]. Other imaging features, such as sarcopenic obesity, aortic calcifications, osteopenia, and mean kidney pixel value, are mentioned as options for frailty assessment [8]. However, an optimal radiological parameter for frailty assessment has still not been selected. Therefore, our study aimed to evaluate the efficacy of various imaging features for frailty assessment in patients undergoing emergency laparotomy.

## 2. Materials and Methods

This study was approved by the local research ethics committee following the guidelines of the Declaration of Helsinki of 1975, with its later amendments. The study obtained the approval of the Ethics Committee of the Jagiellonian University no. 1072.6120.14.2020. Every patient has given informed consent to be included in the study.

A retrospective database of patients who have undergone emergency surgical intervention between January 2016 and December 2020 was developed. A dataset was created in a tertiary referral university hospital, with an annual volume of around 1500 emergency surgeries. Patients that qualified for emergency surgery with preoperative CT scans were included in the study. We excluded patients submitted to the surgery with different imaging modalities used (X-ray, ultrasound) or with no preoperative imaging. Due to the unit profile, only patients above 18 years old were included in the study. Multiple trauma patients were excluded from the analysis. Additionally, every patient had their frailty assessed with the frailty index (mFI) and brief geriatric assessment (BGA) scales [5]. Complications were reported following the Clavien–Dindo classification [14].

All CT studies were acquired on a 64-slice GE Optima CT660 scanner (GE Healthcare, Boston, MA, USA) and included series before and after administering contrast agents. Venous phase series were extracted for further analysis. All volumes had a maximum of 1.3 mm layer height.

The exported images were reviewed using the RadiAnt DICOM viewer (Medixant, Poznań, Poland) and Mimics (Materialise NV, Leuven, Belgium) for the assessment of osteopenia, sarcopenia, sarcopenic obesity, renal volume, and abdominal aorta calcification rate. CT scan measurements were carried out by trained evaluators and reviewed by a board-certified radiologist with 15 years of experience in abdominal and emergency imaging.

**Figure 1:**
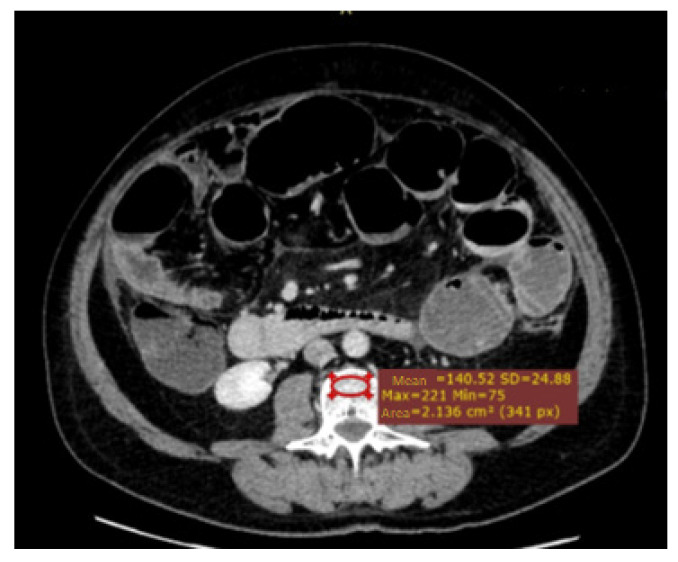
Osteopenia assessment—ROI (region of interest)—anterior trabecular area of the vertebrae on an axial projection at the L3 level.

As described previously, measurement of the attenuation in Hounsfield units (HU) within the region of interest (ROI) is the preferred way to assess osteopenia [15]. A 2D ROI was placed in the anterior trabecular area of the vertebrae on an axial projection at the L3 level (Figure 1).

**Figure 2:**
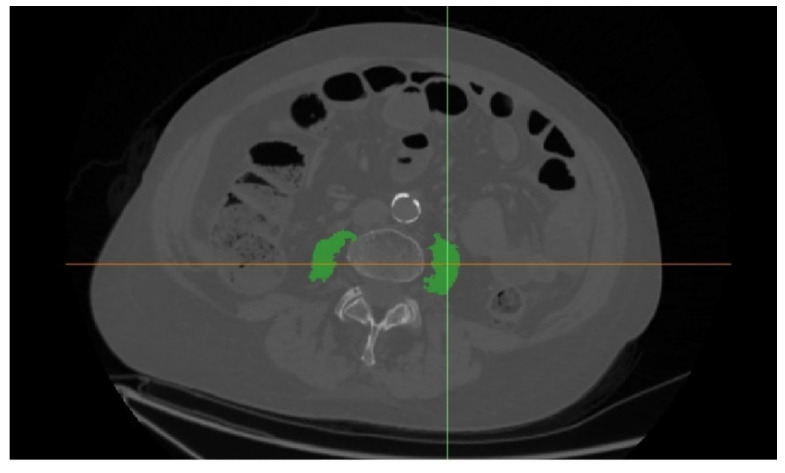
Bilateral psoas muscles area.

Sarcopenia assessment was defined as the bilateral psoas muscles area normalized for the patient’s height (cm^2^/m^2^) at the level of L3. The area was contoured semi-automatically with region growth algorithms and (−29,150 HU) limits. The segmented areas were manually corrected if necessary (Figure 2).

**Figure 3:**
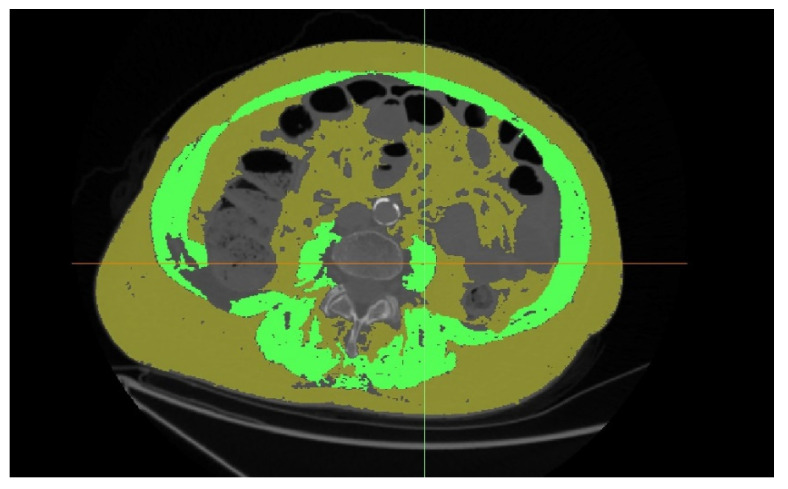
Sarcopenic obesity assessment.

The sarcopenic obesity parameter was defined as the total cross-sectional visceral and subcutaneous fat tissue area divided by the total cross-sectional muscle tissue area on a single axial slice at L3 (Figure 3). Following the approach from other studies, region growth algorithms were used with the (−150, 50 HU) range for visceral fat and (−190, 30 HU) for subcutaneous fat [16].

**Figure 4:**
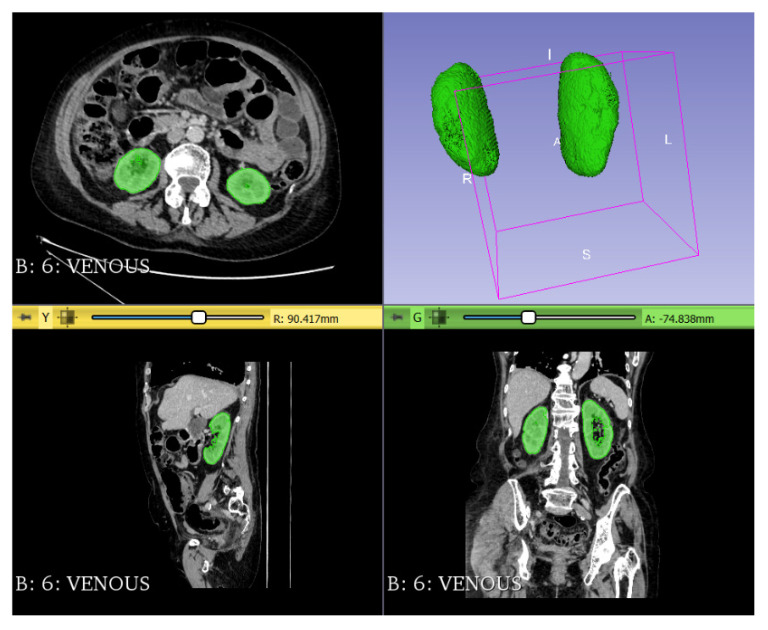
Renal volume measurement.

Kidneys were segmented semi-automatically and bilateral renal volume was normalized for the patient’s height (Figure 4). All artifacts, kidney vessels, renal calyces and pelvises, and renal cysts were manually excluded from the volume calculations.

**Figure 5:**
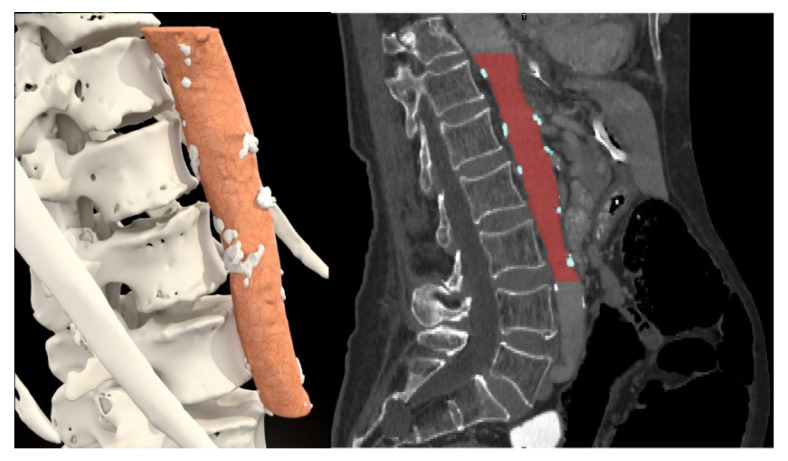
Calcification plaque measurement in aorta.

The abdominal aorta calcification rate was calculated as a percentage of calcification volume divided by the total aorta volume. The aorta was segmented from Th12 to the bottom edge of L3 (Figure 5).

### Statistical Analysis

The primary outcome was the mortality rate. The secondary outcomes were the following perioperative features: complication rate, surgery duration, intensive care unit (ICU) admission, and readmission rate. All data were analyzed with Statistica version 13.0 PL (StatSoft Inc., Tulsa, OK, USA). Continuous results are presented as the median and interquartile range (IQR). Categorical variables were compared by the chi-square test. The Shapiro–Wilk test was used to check for the normal distribution of data, and Student’s *t*-test was used for normally distributed quantitative data. For non-normally distributed quantitative variables, the Mann–Whitney *U* test was used. A *p*-value < 0.05 was considered statistically significant. All considerable patient and treatment-related factors were analyzed with univariate logistic regression models in search of risk factors for mortality and morbidity. Receiver operating curves (ROC) were used to set cut-off points for frailty regarding psoas muscle area/height (PMA), sarcopenic obesity (SO), aortic calcification (AC), osteopenia and kidney volume (KV).

## 3. Results

A total of 261 patients were included in the analysis. The demographic characteristics of the study group are included in Table 1.

A total of 46 patients (17.6%) died within 30 days of surgical intervention and formed group 2. The remaining 215 patients survived and formed group 1. Patients in group 2 were significantly older (77 (63-83 IQR) vs. 63 (47-74 IQR), *p* < 0.001). Moreover, CD III-V complications were significantly more common in group 2 (22.8% vs. 100%, *p* < 0.001), and they were more often admitted to the ICU (17.2% vs. 71.1%, *p* < 0.001). Both mFI and BGA scales identified more high-risk patients in group 2 (*p* < 0.001). The most common operation in both groups was colon resection (30.23% in group 1 and 56.52% in group 2). Numerical values for perioperative outcomes in both groups are presented in Table 2.

The area under the ROC (AUROC) for mortality was the largest for calcification plaques (AUC = 0.736, cut-off point = 0.019, *p* < 0.001). AUROC for osteopenia was 0.725, cut-off point = 100.85, *p* < 0.001. AUROC for the psoas muscle area (PMA) was 0.693, cut-off point = 5.768, *p* < 0.001. AUROC for kidney volume/height was 0.679, cut-off point = 72.158, *p* < 0.001. AUROC for sarcopenic obesity was 0.580, cut-off point = 3.836, *p* = 0.095 (Table 3).

Patients in group 2 had lower median PMA than patients in group 1 (5.19 (4.06–7.39 IQR) vs. 7.16 (5.01–9.41), *p* < 0.001). They also had higher median sarcopenic obesity levels (2.50 (1.62–3.84 IQR) vs. 2.29 (1.23–3.10 IQR), *p* = 0.093). Patients in group 2 had a much higher median percent of atherosclerotic plaques in the aorta volume than patients in group 1 (3.69% (1.87–8.24 IQR) vs. 0.60% (0.0–2.91 IQR), *p* < 0.001). Mean kidney volume/height was higher in group 1 than in group 2 (89.1 vs. 66.7, *p* < 0.001) (Table 4).

Univariate logistic regression revealed the following risk factors for 30-day mortality: every 10 years of age (OR: 1.763, 95%CI: 1.369–2.270, *p* < 0.001), every next ASA class (OR: 6.529, 95%CI: 3.461–12.318, *p* < 0.001), intraoperative adverse events (OR: 9.294, 95%CI: 3.375–25.596, *p* < 0.001), every point of mFI-5 score (OR: 2.485, 95%CI: 1.839–3.357, *p* < 0.001), every point of BGA score (OR: 1.449, 95%CI: 1.219–1.721, *p* < 0.001), PMA (OR: 3.937, 95%CI: 2.023–7.660, *p* < 0.001), sarcopenic obesity (OR: 3.154, 95%CI: 1.388–7.169, *p* = 0.006), osteopenia (OR: 4.658, 95%CI: 2.369–9.157, *p* < 0.001), calcification volume rate in aorta (OR: 6.352, 95%CI: 3.116–13.129, *p* < 0.001) and kidney volume/height (OR: 6.352, 95%CI: 3.201–12.605, *p* < 0.001).

Multivariate logistic regression identified the following risk factors for 30-day mortality: every next ASA class (OR: 4.161, 95%CI: 1.672–10.355, *p* = 0.002), intraoperative adverse events (OR: 12.397, 95%CI: 2.166–70.969, *p* = 0.005) and osteopenia (OR: 4.213, 95%CI: 1.235–14.367, *p* = 0.022) (Table 5).

AUROC for major complications was the largest for aortic calcifications (AUC = 0.688, cut-off point = 2.78, *p* < 0.001). AUROC for major complications for kidney volume/height was 0.629, cut-off point = 75.18, *p* = 0.001. AUROC for major complications for osteopenia was 0.621, cut-off point = 115.34, *p* = 0.001. AUROC for major complications for PMA was 0.617, cut-off point = 10.873, *p* = 0.002. AUROC for major complications for sarcopenic obesity was 0.559, cut-off point = 0.72, *p* = 0.120 (Table 6).

Univariate logistic regression revealed the following risk factors for major complications: every 10 years of age (OR: 1.466, 95%CI: 1.242–1.729, *p* < 0.001), every next ASA class (OR: 3.546, 95%Cl: 2.335–5.387, *p* < 0.001), laparotomy vs. laparoscopy (OR: 3.343, 95%CI: 1.340–8.342, *p* = 0.010), every point of mFI-5 score (OR: 2.107, 95%Cl: 1.644–2.699, *p* < 0.001), every point of BGA score (OR: 1.812, 95%Cl: 1.431–2.281, *p* < 0.001), PMA (OR: 2.408, 95%CI: 1.431–4.053, *p* = 0.001), sarcopenic obesity (OR: 2.843, 95%Cl: 1.323–6.109, *p* = 0.007), osteopenia (OR: 2.813, 95%Cl: 1.568–5.048, *p* = 0.001), calcification volume rate in aorta (OR: 3.805, 95%Cl: 2.222–6.517, *p* < 0.001) and kidney volume/height (OR: 4.594, 95%Cl: 2.627–8.033, *p* < 0.001).

Multivariate logistic regression identified the following risk factors for major complications: every next ASA class (OR: 1.952, 95%Cl: 1.171–3.256, *p* = 0.010) and every point of BGA score (OR: 1.496, 95%Cl: 1.110–2.016, *p* = 0.008) (Table 7).

## 4. Discussion

Our study revealed that osteopenia is the most useful radiological feature for 30-day mortality prediction in patients requiring emergency laparotomy. None of the evaluated imaging markers of frailty proved to be efficient in the prediction of major complication occurrence.

As the number of frail patients requiring surgical interventions increases, methods of its assessment emerge. The modified frailty index was identified as an independent risk factor of postoperative morbidity in patients undergoing common general surgery interventions [4]. Furthermore, as a study by Lee points out, frailty is not only a risk factor for perioperative complications, but also for a negative 1-year prognosis [17]. In our study, we also used mFI and BGA scales for frailty assessment; however, only BGA was useful for the stratification of risk for perioperative complications.

Traditional scoring systems require knowledge of multiple patient-dependent factors. It makes this approach very limited in emergency situations, and quick imaging analysis could help overcome this. Radiological features of frailty are recognized as a predictor of a negative outcome in cardiothoracic procedures [10,18]. Richards et al. drew the same conclusion regarding colorectal cancer surgery, where a CT scan is performed routinely as a part of preoperative staging [9]. Not every patient requiring emergency surgery has a CT scan performed preoperatively. Having said that, patients without CT scans are usually undergoing less complex procedures and are not that susceptible to frailty.

Among the radiological markers of frailty, the most recognizable is the area of psoas major muscle, used to assess sarcopenia. Shinohara et al. noticed a relation between sarcopenia and poor prognosis in patients treated for non-small cell lung carcinoma [11]. In addition, Okamura pointed out its importance in the outcomes of aortic valve replacement [10]. Yamashita et al. identified sarcopenic obesity as a risk factor for poor muscle function, and therefore poor prognosis after cardiovascular surgery [19]. However, most studies concentrate on elective cases [9,10,18]. Gomibuchi used it as a part of the assessment of patients with type A aortic dissection, pointing out the importance of frailty in this procedure [20]. Simpson et al. compared PMA to the P-POSSUM scale in patients over 80 years old undergoing emergency laparotomy and found PMA as a worthy indicator of postoperative mortality [12].

In our study, we tried to find this correlation in emergency cases, broadening the patients’ spectrum to a whole population; however, as was the case for Mccusker et al., who did not find this relation in geriatric trauma patients, we were also unable to indicate it [21]. Anastácio et al. point out that volumetric measurements, in addition to the composition of the body, might represent the signs of frailty that underline the meaning of sarcopenic obesity [13]. Data regarding the impact of sarcopenic obesity on abdominal surgery are limited and mostly focused on pancreatic and gastric cancer [22,23,24,25]. On the other hand, Kaplan et al. indicated that both osteopenia and sarcopenia were independently associated with increased 1-year mortality in 65-year-old and older patients admitted to ICU after traumatic injury [15].

Aortic calcifications and kidney volume were not identified as risk factors either for 30-day mortality or for morbidity. However, only a few papers assessed these factors. There is strong evidence that they might be useful for frailty assessments.

Our study has some limitations. Firstly, our analysis is retrospective and requires investigation in a larger, prospective scenario. Moreover, due to the nature of the study, we included only patients with available CT scans obtained preoperatively. This caused the exclusion of patients with a better overall condition and milder diseases, such as appendicitis. As no CT is required in these cases, one might suspect that patients in that group would be less prone to frailty. Furthermore, a CT scan is time-consuming, which limits the utilization of radiological frailty assessment in unstable, trauma patients. Finally, although our approach overcomes the problem of lacking patient’s medical information, this method is time-consuming and requires a radiologist or a skilled evaluator to perform measurements. This, however, will be automatized in the future with advances in medical image processing.

## 5. Conclusions

Osteopenia was the best parameter for perioperative mortality risk stratification in patients undergoing emergency surgical intervention. Sarcopenia (measured as psoas muscle area), sarcopenic obesity, aortic calcifications and mean kidney volume do not predict poor outcomes in those patients. None of the radiological markers appeared to be useful in the prediction of perioperative morbidity.

## Figures and Tables

**Table 1:** Baseline characteristics of study groups.

Parameter	Total	Group 1 (30-Day Mortality—0)	Group 2 (30-Day Mortality—1)	*p* Value
Patients, *n* (%)	261	215 (82.4%)	46 (17.6%)	-
Females, *n* (%)	128 (49%)	105 (48.8%)	23 (50%)	0.886
Median age (IQR) (years)	65 (52–76)	63 (47–74)	77 (63–83)	<0.001
Median BMI (IQR) (kg/m^2^)	25.33 (22.86–28.22)	24.91 (22.67–28.08)	25.83 (23.00–29.29)	0.172
ASA class: I, *n* (%) II, *n* (%) III, *n* (%) IV, *n* (%) V, *n* (%)	25 (9.6%) 88 (33.7%) 92 (35.2%) 52 (19.9%) 4 (1.5%)	25 (11.6%) 87 (40.5%) 76 (35.3%) 27 (12.6%) 0 (0%)	0 (0%) 1 (2.2%) 16 (34.8%) 25 (54.3%) 4 (8.7%)	<0.001
Comorbidities: Hypertension, *n* (%) Diabetes, *n* (%) Heart failure, *n* (%) COPD or recent pneumonia, *n* (%) End stage renal failure, *n* (%)	107 (41.0%) 47 (18.0%) 54 (20.7%) 21 (8.0%) 7 (2.7%)	80 (37.2%) 30 (14.0%) 32 (14.9%) 14 (6.5%) 2 (0.9%)	27 (58.7%) 17 (37.0%) 22 (47.8%) 7 (15.2%) 5 (10.7%)	0.007 <0.001 <0.001 0.049 <0.001
mFI scale: Low risk, *n* (%) Intermediate risk, *n* (%) High risk, *n* (%) Unable to assess, *n* (%)	114 (43.7%) 61 (23.4%) 80 (30.7%) 6 (2.3%)	107 (49.8%) 54 (25.15%) 51 (23.7%) 3 (1.4%)	7 (15.2%) 7 (15.2%) 29 (63.0%) 3 (6.5%)	<0.001
BGA scale: Low risk, *n* (%) Intermediate risk, *n* (%) High risk, *n* (%) Unable to assess, n (%)	214 (82.0%) 13 (5.0%) 10 (3.8%) 24 (9.2%)	203 (94.4%) 7 (3.3%) 5 (2.3%) 0 (0%)	11 (23.9%) 6 (13.0%) 5 (10.9%) 24 (52.2%)	<0.001
Type of surgery: Appendectomy, *n* (%) Cholecystectomy, n (%) Colon resection, *n* (%) Small bowel resection, *n* (%) Laparotomy without resection, *n* (%) Ulcer perforation surgery, *n* (%) Other, *n* (%)	33 (12.64%) 15 (5.75%) 91 (34.87%) 40 (15.33%) 41 (15.7%) 25 (9.58%) 16 (6.13%)	33 (15.34%) 15 (6.98%) 65 (30.23%) 32 (14.88%) 37 (17.21%) 20 (9.31%) 13 (6.05%)	0 (0%) 0 (0%) 26 (56.52%) 8 (17.39%) 4 (8.7%) 5 (10.87%) 3 (6.52%)	<0.001

IQR: interquartile range; ASA: physical status classification system; COPD: chronic obstructive pulmonary disease; mFI: modified frailty index; BGA: brief geriatric assessment

**Table 2:** Perioperative outcomes of study groups.

Parameter	Total	Group 1 (30-Day Mortality—0)	Group 2 (30-Day Mortality—1)	*p* Value
Laparoscopy vs. laparotomy, *n*	37 (14.2%) vs. 224 (85.8%)	36 (16.7%) vs. 179 (83.3%)	1 (2.2%) vs. 45 (97.8%)	0.019
Surgeon attending vs. surgery resident operating, *n*	145 (55.6%) vs. 116 (44.4%)	119 (55.3%) vs. 96 (44.7%)	26 (56.5%) vs. 20 (43.5%)	0.959
Median surgery duration (IQR) (minutes)	120 (85–165)	120 (80–165)	135 (90–228)	0.057
Intraoperative adverse events, *n*	18 (6.9%)	7 (3.3%)	11 (23.9%)	<0.001
Median length of hospital stay (IQR) (days)	7 (4–11)	7 (5–11)	4.5 (1–10)	<0.001
Number of patients with admission to ICU	70 (26.8%)	37 (17.2%)	33 (71.1%)	<0.001
Median ICU stay if occurred (IQR) (days)	9 (0–21)	10 (0–28)	7 (2–14)	0.568
Major postoperative complication (Clavien–Dindo scale > 2)	95 (36.4%)	49 (22.8%)	46 (100%)	<0.001

**Table 3:** ROC curves for 30-day mortality.

Parameter/Medical Condition	AUROC	95%CI AUROC	*p* Value
Calcification plaques	0.736	0.659–0.813	<0.001
Osteopenia (ROI at L3)	0.725	0.647–0.804	<0.001
Psoas muscle area/height	0.693	0.610–0.777	<0.001
Kidney volume/height	0.679	0.585–0.773	<0.001
Sarcopenic obesity	0.580	0.486-0.673	0.095

ROC: receiver operating curves; AUROC: the area under the ROC; ROI: the region of interest

**Table 4:** Radiological frailty parameters in study groups.

Parameter	Total	Group 1 (30-Day Mortality—0)	Group 2 (30-Day Mortality—1)	*p* Value
Median psoas muscle area/height (IQR) (cm^2^/m)	7.16 (5.01–9.41)	7.61 (5.38–9.70)	5.19 (4.06–7.39)	<0.001
Number of patients with psoas area/height under cut-off point	94 (36.0%)	65 (30.2%)	29 (63.0%)	<0.001
Median sarcopenic obesity (IQR) (cm^2^/cm^2^)	2.30 (1.33–3.19)	2.29 (1.23–3.10)	2.50 (1.62–3.84)	0.093
Number of patients with sarcopenic obesity under cut-off point	31 (11.9%)	20 (9.3%)	11 (24.4%)	0.004
Median osteopenia in ROI at L3 (IQR) (HU)	138.1 (102.1–181.0)	144.0 (109.6–193.8)	100.6 (74.6–142.5)	<0.001
Number of patients with osteopenia under cut-off point	61 (23.4%)	38 (17.7%)	23 (50.0%)	<0.001
Median percent of atherosclerotic plaques in aorta volume (IQR) (%)	0.86 (0–3.81)	0.60 (0–2.91)	3.69 (1.87–8.24)	<0.001
Number of patients with percent of calcification plaques in aorta volume under cut-off point	100 (38.3%)	66 (30.7%)	34 (73.9%)	<0.001
Mean kidney volume/height ± SD (cm^3^/m)	85.7 (69.7–104.5)	89.1 (73.6–105.7)	66.7 (53.6–93.6)	<0.001
Number of patients with kidney volume/height under cut-off point	79 (30.27)	49 (22.8%)	30 (65.2%)	<0.001

HU: Hounsfield units.

**Table 5:** Risk factors for 30-day mortality.

Parameter	OR	95%CI	*p* Value
Univariate analysis:			
Male sex	0.955	0.505–1.805	0.886
Every 10 years of age	1.763	1.369–2.270	<0.001
Every next ASA class	6.529	3.461–12.318	<0.001
Body mass index (every 1 kg/m^2^)	1.060	0.993–1.130	0.079
Laparotomy vs. laparoscopy (laparotomy-1)	9.050	1.208–67.798	0.032
Surgeon specialist vs. surgeon resident operating (surgeon resident-1)	1.017	0.533–1.942	0.959
Intraoperative adverse events	9.294	3.375–25.596	<0.001
Every point of mFI-5 score	2.485	1.839–3.357	<0.001
Every point of BGA score	1.449	1.219–1.721	<0.001
Psoas muscle area/height under cut-off point	3.937	2.023–7.660	<0.001
Sarcopenic obesity under cut-off point	3.154	1.388–7.169	0.006
Osteopenia under cut-off point	4.658	2.369–9.157	<0.001
Calcification volume rate in aorta under cut-off point	6.396	3.116–13.129	<0.001
Kidney volume/height under cut-off point	6.352	3.201–12.605	<0.001
Multivariate analysis:			
Every 10 years of age	1.098	0.659–1.827	0.720
Every next ASA class	4.161	1.672–10.355	0.002
Intraoperative adverse events	12.397	2.166–70.969	0.005
Every point of mFI-5 score	1.447	0.864–2.424	0.160
Every point of BGA score	1.161	0.905–1.488	0.241
Psoas muscle area/height under cut-off point	2.485	0.781–7.906	0.123
Sarcopenic obesity under cut-off point	1.812	0.469–6.993	0.389
Osteopenia under cut-off point	4.213	1.235–14.367	0.022
Atherosclerosis in aorta under cut-off point	1.241	0.342–4.506	0.743
Kidney volume/height under cut-off point	1.012	0.296–3.464	0.984

**Table 6:** ROC curves for major complications.

Parameter/Medical Condition	AUROC	95%CI AUROC	*p* Value
Calcification plaques	0.688	0.619–0.757	<0.001
Osteopenia	0.621	0.551–0.691	0.001
Psoas muscle area/height	0.617	0.545–0.689	0.002
Kidney volume/height	0.629	0.553–0.704	0.001
Sarcopenic obesity	0.559	0.485–0.632	0.120

**Table 7:** Risk factors for major complications.

Parameter	OR	95%CI	*p* Value
Univariate analysis:			
Male sex	1.007	0.607–1.669	0.980
Every 10 years of age	1.466	1.242–1.729	<0.001
Every next ASA class	3.546	2.335–5.387	<0.001
Body mass index (every 1 kg/m^2^)	1.039	0.985–1.095	0.163
Laparotomy vs. laparoscopy (laparotomy-1)	3.343	1.340–8.342	0.010
Surgeon specialist vs. surgeon resident operating (surgeon resident-1)	0.827	0.496–1.378	0.466
Intraoperative adverse events	3.902	1.414–10.774	0.009
Every point of the mFI-5 score	2.107	1.644–2.699	<0.001
Every point of the BGA score	1.812	1.439–2.281	<0.001
Psoas muscle area/height under cut-off point	2.408	1.431–4.053	0.001
Sarcopenic obesity under cut-off point	2.843	1.323–6.109	0.007
Osteopenia under cut-off point	2.813	1.568–5.048	0.001
Calcification volume rate in aorta under cut-off point	3.805	2.222–6.517	<0.001
Kidney volume/height under cut-off point	4.594	2.627–8.033	<0.001
Multivariate analysis:			
Every 10 years of age	0.997	0.741–1.342	0.987
Every next ASA class	1.952	1.171–3.256	0.010
Every point of the mFI-5 score	1.129	0.748–1.703	0.563
Every point of the BGA score	1.496	1.110–2.016	0.008
Psoas muscle area/height under cut-off point	1.341	0.561–3.207	0.509
Sarcopenic obesity under cut-off point	2.603	0.780–8.691	0.120
Osteopenia under cut-off point	1.262	0.473–3.371	0.642
Atherosclerosis in aorta under cut-off point	1.504	0.606–3.734	0.379
Kidney volume/height under cut-off point	1.963	0.843–4.572	0.118

## Data Availability

The data used for this study are available on demand after contact with the corresponding author.
